# Loco-regional adjuvant radiation therapy in breast cancer patients with positive axillary lymph-nodes at diagnosis (CN2) undergoing preoperative chemotherapy and with complete pathological lymph-nodes response. Development of GRADE (Grades of recommendation, assessment, Development and Evaluation) recommendation by the Italian Association of radiation therapy and Clinical Oncology (AIRO)

**DOI:** 10.1016/j.breast.2020.12.012

**Published:** 2021-01-02

**Authors:** Lorenza Marino, Valentina Lancellotta, Pierfrancesco Franco, Icro Meattini, Bruno Meduri, Marco Bernini, Alessandra Fabi, Renzo Corvò, Stefano M. Magrini, Giovanni L. Pappagallo, Stefano Arcangeli, Rolando M. D’Angelillo

**Affiliations:** a Radiotherapy Oncology Department, Humanitas-Istituto Clinico Catanese, Misterbianco (CT), Italy; b Fondazione Policlinico Universitario A. Gemelli IRCCS, UOC di Radioterapia, Dipartimento di Scienze Radiologiche, Radioterapiche Ed Ematologiche, Roma, Italy; c Department of Oncology, Radiation Oncology, School of Medicine, University of Turin, Turin, Italy; d Department of Experimental and Clinical Biomedical Sciences "M. Serio", University of Florence, Florence, Italy; e Radiation Oncology Unit - Oncology Department, Azienda Ospedaliero Universitaria Careggi, Florence, Italy; f Radiation Oncology Unit, University Hospital of Modena, Modena, Italy; g Breast Surgery Unit, Oncology Department, Azienda Ospedaliero-Universitaria Careggi, University of Florence, Florence, Italy; h Oncology Unit 1, Regina Elena National Cancer Institute, Rome, Italy; i La Sapienza University, Rome, Italy; j Department of Radiation Oncology, IRCCS Ospedale Policlinico San Martino and Department of Health Science, University of Genoa, Genoa, Italy; k Department of Radiation Oncology, ASST Ospedali Civili and Brescia University, Brescia, Italy; l Medical Oncology Unit, Azienda ULSS 3 Serenissima, Mirano-Dolo, Verona, Italy; m Department of Radiation Oncology, Policlinico S. Gerardo and University of Milan "Bicocca", Milan, Italy; n Radiotherapy, Dipartimento di Biomedicina e Prevenzione, Università Degli Studi di Roma Tor Vergata, Rome, Italy

**Keywords:** Breast cancer, Loco-regional radiation therapy, Preoperative chemotherapy

## Abstract

**Objective:**

To perform a meta-analysis to determine the effect of loco-regional radiation therapy (RT) compared to no loco-regional RT for operated patients in clinical stage cN2 breast cancer at diagnosis and ypN0 after preoperative chemotherapy (PST).

**Material and Methods:**

Eligible studies were identified through a systematic search of the medical literature performed independently by two researchers using a validated search strategy. An electronic search of Medline via PubMed and Embase (Breast cancer AND preoperative chemotherapy AND radiation therapy) was conducted with no language or publication status restrictions. The effect of loco-regional RT on overall (OS), disease free (DFS), loco-regional recurrence-free (LRRFS) survival and local recurrence was evaluated. An electronic search of Medline via PubMed and Embase (Toxicity AND radiation therapy breast cancer AND preoperative therapy; toxicity AND breast surgery AND preoperative chemotherapy) was conducted for outcomes of harm: major acute and late skin toxicity, lymphedema and cardiac events.

**Results:**

Of 333 studies identified, 4 retrospective studies reporting on a total of 1107 patients were included in the meta-analysis. Six and 3 reported data of acute and late skin toxicity, while 2 studies provided information on cardiac events. Pooled results showed no difference in terms of hazard ratio for loco-regional RT versus no loco-regional RT [hazard ratio (HR) = 0.82, 95% confidence interval (CI) 0.63–1.68]. Loco-regional RT was associated with an OS benefit in the subgroup analysis: IIIB-C (loco-regional RT 79.3% *vs* no loco-regional RT 71.2%, *p = 0.027*) and T3-T4 (loco-regional RT 82.6% *vs* no loco-regional RT 76.6%, *p = 0.025*). No difference was shown in terms of 5-year DFS (loco-regional RT 91.2% *vs* no loco-regional RT 83%, *p = 0.441*) and LRRFS (loco-regional RT 98.1% *vs* no loco-regional RT 92.3%, *p = 0.148*). There was no significant difference between the groups in terms of acute and late skin toxicities, lymphedema and cardiac events.

**Conclusions:**

Because of the limitations due to the small number of studies and heterogeneity in the analysis, the present study does not allow to draw any definitive conclusion, highlighting the need for well-controlled trials to determine the effect of loco-regional RT in patients with cN2 having a pathological complete response in the axillary nodes after preoperative chemotherapy.

## Highlights

• The prognostic impact of pCR after primary systemic therapy on DFS and OS has been shown in meta-analyses of randomized phase III trials.
• The association of treatment response with loco-regional recurrence has been studied only in retrospective reports.
• RNI should be strongly considered in patients with clinically involved lymph nodes regardless of the response to primary systemic therapy, especially in the presence of further risk factors.
• In patients with cN2 at diagnosis and ypN0 at surgery after PST, loco-regional RT should be evaluated for each patient in the multidisciplinary team.

## 1 Introduction

Preoperative chemotherapy (PST) in breast cancer (BC) patients was historically used in locally advanced disease. More recently, it is increasingly being applied in earlier stage BC, although no advantage in survival was observed as compared to postoperative systemic therapy [[1], [2], [3], [4]]. Several studies showed a survival benefit in patients with pathological complete response (pCR), except for luminal A-patients [5,6] suggesting that the response to PST may have clinical implications such as a higher chance to offer breast conserving therapy [7,8] and a less extensive treatment of the axilla, if after PST, tumor-positive (axillary) lymph nodes are converted into ypN0 [7,[9], [10], [11], [12]]. It means that it is possible to modulate postoperative treatments according to the response to PST, for specific biological types. An example could be intensification of postoperative treatment with TDM1 for Stage I –II, HER2 positive (HER2+) disease achieving less than pCR after PST [13]. Randomized trials have established that administration of loco-regional radiation therapy (RT) to appropriately selected women who receive postoperative chemotherapy after mastectomy reduces loco-regional recurrence and breast cancer mortality [[13], [14], [15], [16]]. The large grey area includes the role of loco-regional irradiation in breast cancer patients with cN2 at diagnosis and achieving a pCR in the axillary nodes (ypN0) after PST because no randomized trials have been performed to define which patients benefit from loco-regional RT after PST.

Pathologic downstaging might include complete response of the primary tumor and/or axillary disease and thus the conventional role of loco-regional RT may not be clear [17,18]. Several studies showed that the recurrence risk is influenced by tumor biology and by both the pre-and post PST tumor stage [[19], [20], [21], [22], [23], [24], [25]] suggesting the use of loco-regional RT in patients with ≥cT3N + disease, or patients with ≥cT1-4N2 disease regardless of their response to PST [26]. An analysis of the National Surgical Adjuvant Breast and Bowel Project (NSABP) B-18 and B-27 PST trials identified initial clinical stage and response to therapy as significant predictors for local-regional recurrence (LRR) patients receiving mastectomy, none of whom received loco-regional RT [27]. Although pCR has been associated with improved survival, the association is stronger in triple-negative and HER2+ hormone receptor (HR)-negative patients who received an anti-HER2 therapy [28,29]. Therefore, recommendations for loco-regional RT in specific subtypes could result in overtreatment [30,31].

Due to these uncertanties, the Association of Radiation and Clinical Oncology (AIRO) Guidelines decided to develop a clinical recommendation on this topic (patients with breast cancer cN2 at diagnosis turning ypN0 after preoperative chemotherapy), based on the Grades of Recommendation, Assessment, Development, and Evaluation (GRADE) approach.

## 2 Materials and methods

### 2.1 Development of clinical questions

The clinical question was developed following the P.I.C.O. acronym, as follow: population (P), intervention (I), comparison (C), and outcomes (O). For the 2019 version of AIRO guidelines on breast cancer, the panel expressed the following clinical question:

(P) In operated breast cancer patients with cN2 at diagnosis and ypN0 after PST, is loco-regional RT (I) superior when compared to no loco-regional RT (C), in relation to the outcomes (O) of benefit and harm. Development of GRADE (Grades of Recommendation, Assessment, Development and Evaluation) Recommendation by the Italian Association of radiation therapy and Clinical Oncology (AIRO).

### 2.2 Identification of outcomes

The panel identified the following outcomes of benefit: overall (OS), breast cancer specific (BCSS), disease free survival (DFS) and local control (LC). The following outcomes of harm were identified by the panel: acute and late skin toxicities, lymphedema, lung and heart grade 3–4 toxicities. All these outcomes were considered as “critical” for the decision-making process.

### 2.3 Search strategy and selection of evidence

This systematic review was conducted in accordance with the PRISMA guidelines [32]. We performed a comprehensive literature search using PubMed and Embase (up to July 2019) to identify the full articles evaluating the efficacy and the safety of loco-regional RT in breast cancer patients with cN2 at diagnosis and turning ypN0 after neo-postoperative chemotherapy. ClinicalTrials.gov was searched for ongoing or recently completed trials, and PROSPERO was searched for ongoing or recently completed systematic reviews. Electronic searches were supplemented by manually searching the references of included studies and review articles.

The studies were identified using the following medical subject headings (MeSH) and keywords including “breast neoplasms”, “preoperative chemotherapy”, “postoperative radiation therapy”, “toxicity”. The Medline search strategy was (“breast neoplasia” [Mesh] OR “breast neoplasia” [All fields]) AND (“preoperative therapy” [Mesh] OR “preoperative therapy” [All fields]), AND “radiation therapy” [Mesh] OR “radiation therapy” [All fields]) AND “toxicity” [Mesh] OR “toxicity” [All fields]). The search was restricted to English language.

We analyzed only clinical studies presented as full texts and investigating the population of breast cancer patients with cN2 at diagnosis and ypN0 after PST, who underwent loco-regional RT.

Conference papers, surveys, letters, editorials, book chapters, and reviews were excluded. Time restriction (1990–2019) of the publication was considered.

Studies were identified on a search process performed by two independent reviewers (VL, LM), and uncertainty regarding eligibility was resolved by consulting a third reviewer. Eligible citations were retrieved for full-text review. Finally, a committee including members of the AIRO Task Force performed an independent check and the definitive approval of the review.

Inclusion criteria for the meta-analysis were: (1) randomized-controlled trials (RCTs), prospective, retrospective, and cohort studies; (2) utilization of PST in cN2; (3) patients receiving or not postoperative RT; (5) reported quantitative outcome data. The following information and data were extracted from studies that met the inclusion criteria: the name of the first author, year of publication, study design, number of patients in each group and outcome data.

The meta-analysis was conducted in accordance with the recommendations made by the Cochrane Collaboration as well as the PRISMA statement. Hazard ratio (HR) was used as effect measure for time-to-event outcomes [OS and DFS] in two studies. Meta-analysis was conducted using a random effects model. We assessed statistical heterogeneity with I^2^ statistics. This method describes the proportion of total variation between included studies that are attributable to differences between studies rather than sampling error (chance). Statistical heterogeneity between groups was considered relevant for comparisons with I^2^ statistics of >50%.

The GRADEpro Guideline Development Tool (GDT) was used to create Summary of Findings (SoF) tables in Cochrane systematic reviews.

### 2.4 Quality of evidence evaluation

Certainty of evidence for all selected outcomes was performed according to the GRADE approach, considering study limitations, imprecision, indirectness, inconsistency and publication bias. Certainty level starts at higher pre-specified level for randomized controlled trials, but levels of certainty can be downgraded if limitations in one of the abovementioned domains are detected. Evidence can be classified as high, moderate, low and very low level of certainty.

### 2.5 Benefit/harm balance and clinical recommendation

Based on the summary of evidence, the panel expressed one of the following judgments about the benefit to risk ratio between intervention and comparison: favorable, uncertain/favorable, uncertain (both for intervention or comparison), uncertain/unfavorable, and unfavorable. The strength of the recommendation could be considered as strong positive, conditional positive, uncertain, conditional negative or strong negative.

## 3 Results

### 3.1 Search strategy results and details of the identified relevant studies

**Fig. 1:**
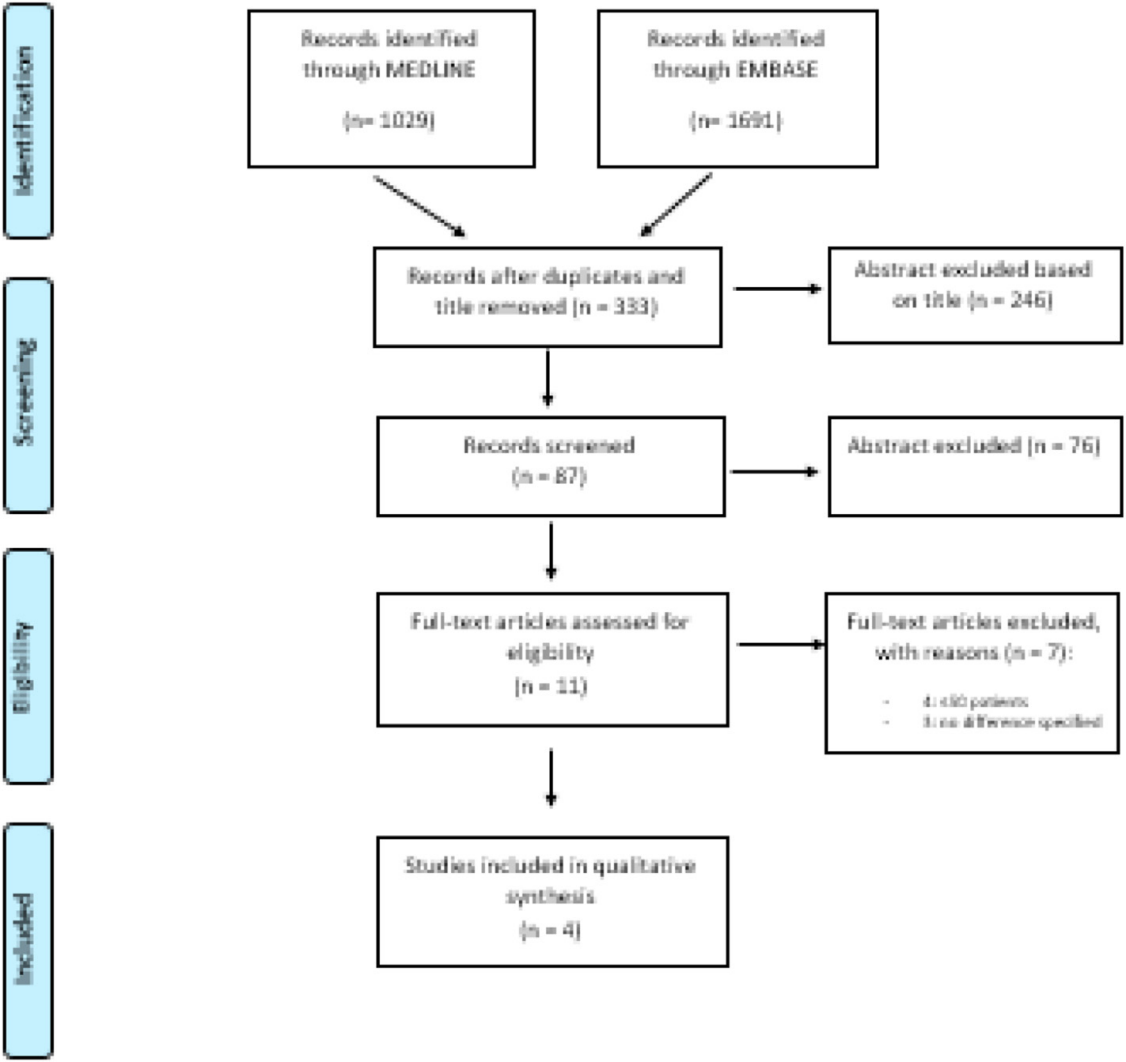
PRISMA (Preferred Reporting Items for Systematic Reviews and Meta-Analyses) Flow chart for outcomes of benefit.

A flow diagram of study selection for primary outcomes (benefit outcomes) is shown in Fig. 1. A total of 333 potentially relevant studies was identified through the database searches after duplicates removal. These articles were screened by title and abstract and 246 were excluded. Of these, 83 articles were excluded because of few numbers of patients (<50 patients) and lack of comparison group (Fig. 1), leaving four studies for the analysis [31,[33], [34], [35]].

The retrospective study of Kantor et al. evaluated the role of loco-regional RT in the subgroup of patients that were ypN0 after PST [33]. About 698 patients (8.4%) had a complete response to PST in the breast and 1937 (23.3%) had tumor-negative axillary nodes after PST. Four hundred-six patients were cN2, of these 41 (40.1%) received ENI while 32 patients (20.1%) underwent exclusive surgery.

The retrospective study of Liu et al. [34] evaluated the role of loco-regional RT after mastectomy vs non-loco-regional RT on a cohort of 251 breast cancer patients with cN2 at diagnosis and ypN0 after PST. Of the 251 cN2 patients, 90 (of n = 657) (13.1%) underwent loco-regional RT and 161 (of n = 903) (17.8%) did not. Irradiated patients had less comorbidities; more advanced stage, a higher number of regional lymph nodes examined, and less unknown ER/PR status, and were more likely to receive multi-agent chemotherapy or endocrine therapy. Target volumes included chest wall and lymph nodes, with or without a boost to the chest wall. The median RT dose was 50.4 Gy.

The KROG 12-05 [35] study is a retrospective trial that evaluated the role of loco-regional RT in patients undergoing mastectomy *vs* non-loco-regional RT on a sample of 151 breast cancer patients with cN2 at diagnosis. The most common PST regimen was a combination of anthracycline and taxane (55.6%), followed by anthracycline-based (36.4%) and taxane-based (6.0%) chemotherapy. Three patients (2%) received a different regimen. All patients underwent mastectomy. Complete axillary lymph node dissection was performed in 141 patients (93.4%) and sentinel lymph node biopsy alone was performed in 10 (6.7%). Seventy-two (47.6%) and 19 patients (12.6%) received postoperative hormonal therapy and targeted therapy, respectively. The radiation target volumes were the chest wall and regional lymph-nodes (axilla and supraclavicular fossa, with or without the internal mammary chain). A total RT dose of 45–50 Gy was delivered.

The KROG 16-16 study attempted to investigate the potential subgroups (as cN2 and cT3-4) that would benefit ENI, in ypN0 as after PST and breast-conserving surgery [31]. Seventy-three patients were cN2, of these 41 (40.1%) received ENI while 32 patients (20.1%) did not. The most frequent PST regimen was anthracycline plus taxane (41.0%), followed by anthracycline with cyclophosphamide combined with taxane (32.2%) and anthracycline in combination with cyclophosphamide (11.5%). Axillary lymph-nodes (LN) fine-needle aspiration before PST was performed in 107 patients (41.0%). Axillary LN dissection (ALND) was performed in 213 patients (81.6%), while 48 patients (18.4%) received sentinel LN biopsy (SLNB) alone. Postoperative hormone treatment was administrated to 86 patients (33.0%). HER2-targeted therapy was offered to 48 patients (50.2%).

The postoperative RT dose to the whole breast was 45–54 Gy given in 1.8–2.0 Gy per fraction. Boost RT to the primary tumor bed was delivered in 233 patients (89.3%). ENI including the supraclavicular region was performed in 102 patients (39.1%), of whom 20 (7.7%) received internal mammary irradiation. The RT dose for ENI was 45–54 Gy given in conventional fractionation.

**Fig. 2:**
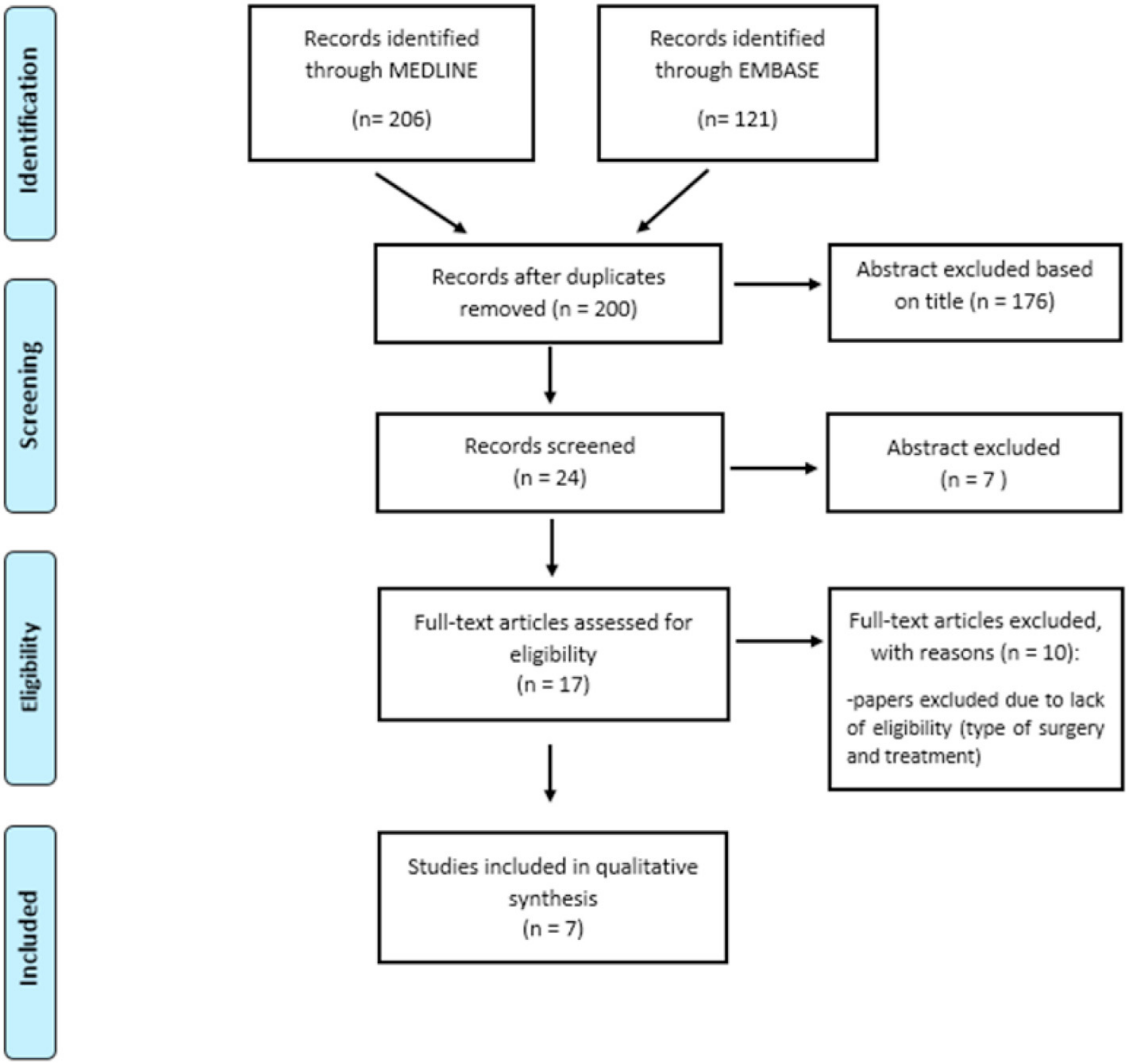
PRISMA (Preferred Reporting Items for Systematic Reviews and Meta-Analyses) Flow chart for outcomes of harm.

A flow diagram regarding the study selection for outcomes of harm is shown in Fig. 2. A total of 200 potentially relevant studies were identified through the database searches after duplicates were removed. These articles were screened by title and abstract and 176 were excluded. The remaining full-text articles were reviewed and 17 were excluded, the reasons for which are shown in Fig. 2. Thus, ultimately, seven articles were included in the meta-analysis [31,[37], [38], [39], [40], [41], [42]] and all studies were retrospective.

### 3.2 Outcomes of benefit

All the four identified studies reported OS rates. Three out of four studies showed that loco-regional RT in breast cancer patients with cN2 at diagnosis who experience ypN0 after PST did not improve OS [31,33,35] while only Liu et al. showed a significant advantage for loco-regional RT in patients with breast staged as IIIB-IIIC or T3/T4 (HR for patients receiving RT 0.82 (0.63–1.068).

Only two studies reported CSS rates, showing that loco-regional RT did not improve CSS [31,35].

The LC rates were reported in two studies that showed loco-regional RT not to improve LC [35,36].

Finally, only two studies reported DFS rates showing that loco-regional RT did not improve it [35,36].

The Summary of Findings (SoF) tables for outcomes of benefit was reported in Table 1.

**Table 1:** Summary of findings (SoF) table for outcomes of benefit.

Study design	Risk of bias	Inconsistency	Indirectness	Imprecision	N. of patients		Effect		Certainty
					PMRT	no PMRT	Relative (95% CI)	Absolute (95% CI)	
OS (Shim, Int J Radiation Oncol Biol Phys 2014) (follow-up: median 59 months; assessed with: events)									
Observational	Not serious	Not serious	Serious^a^	Serious^b^	5-years OS: RT: 93.3% vs no RT 89.9% (p = 0.443)				Very low
OS (Liu, Oncotarget, 2015) (follow-up: median 56 months; assessed with: HR)									
Observational	Not serious	Not serious	Serious^c^	Serious^d^	HR for RT: 0.82 (0.63–1.068) SUB-GROUP ANALYSIS: 5-years OS IIIB-IIIC: RT 79.3% vs no RT 71.2% (p = 0.027); 5-years OS T3-T4: RT: 82.6% vs no RT 76.6% (p = 0.025)				Very low
OS (Cho, Clinical Breast Cancer, 2019) (follow-up median 79 months)									
Observational	Not serious	Not serious	Serious^a^	Serious^d^	HR for RT: 0.350 (0.096–1.272)				Very low
OS (Kantor, J Surg Oncol, 2017) (follow-up: median 69 months; assessed with: HR)									
Observational	Serious^e^	Not serious	Not serious	Not serious	5-years OS: RT 92.9% vs no RT 83% (p = 0.441)				Very low
DFS (Shim, Int J Radiation Oncol Biol Phys 2014) (follow-up: median 59 months; assessed with: KM)									
Observational	Not serious	Not serious	Serious^a^	Serious^b^	5-years DFS: RT 91.2% vs no RT 83% (p = 0.441)				Very low
DFS (Cho, Clinical Breast Cancer, 2019) (follow-up median 79 months; assessed with: HR)									
Observational	Not serious	Not serious	Serious^a^	Serious^d^	HR: 0.561 (0.249–1.264)				Very low
LRRFS (Shim, Int J Radiation Oncol Biol Phys 2014)									
Observational	Not serious	Not serious	Serious^a^	Serious^b^	5-years LRRFS: RT 98.1% vs no RT 92.3% (p = 0.148)				Very low
LRRFS (Cho, Clinical Breast Cancer, 2019) (follow-up median 79 months; assessed with: HR)									
Observational	Not serious	Not serious	Serious^a^	Serious^d^	HR: 0.310 (0.148–1.833)				Very low

*Abbreviations:* RT: radiotherapy; OS: overall survival; DFS: disease free survival; LRRFS: loco-regional recurrence-free; HR: hazard ratio; CI: confidence interval; RR: risk ratio; PST: preoperative chemotherapy.

Explanations.

a Indirectness for population, included both cN1 and cN2 diagnosis downstaged to ypN0 after PST.

b Small population.

c Indirectness for population, included both in Stage II and III.

d CI both included effect and no effect.

e Possible selection bias due to a sub-group analysis of large database.

The certainty of evidence was judged as “very low” for each outcomes of benefit for the following reasons: indirectness for population including both cN1 and cN2 [35,36] or stage II and II [34], imprecision for small population [35] or CI including both effect and no-effect [35,36] or finally to possible selection bias due to a sub-group analysis of a large database [33].

### 3.3 Outcomes of harm

Six studies reported data on acute skin toxicity (grade 3 and 4) [[36], [37], [38], [39],^41^,^42^]. Three studies reported data of late skin toxicity (grade 3 and 4), lymphedema and late lung toxicity [36,40,42].

**Fig. 3:**
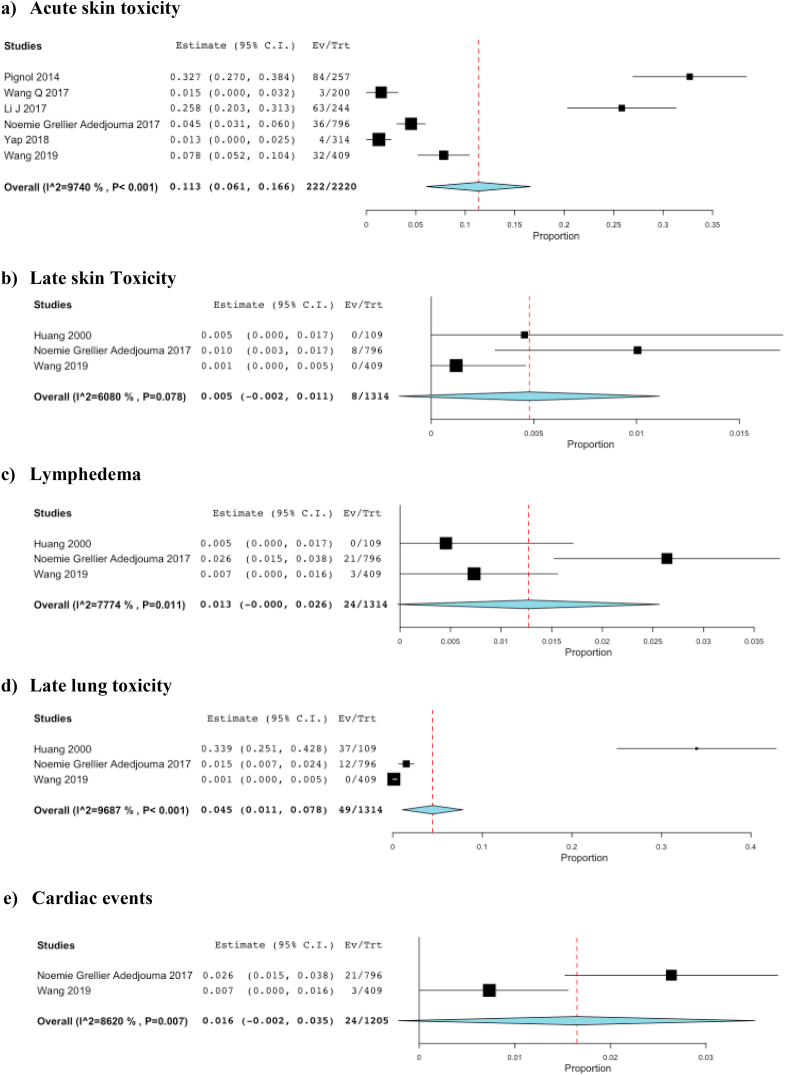
Forest Plots for Outcomes of Harm. a. Acute skin toxicity. b. Late skin toxicity. c. Lymphoedema. d. Late lung toxicity. e. Cardiac events.

Significant heterogeneity was found for acute skin toxicity with 222 events, pooled analysis 11% (95% CI 6.1–17%, I^2^ = 97%) (Fig. 3a), late skin toxicity with 8 events, pooled analysis 0.5% (95% CI 0.1–1%, I^2^ = 61%) (Fig. 3b), lymphedema with 24 events, pooled analysis 1.3% (95% CI 0.2–6%, I^2^ = 78%) (Fig. 3c) and late lung toxicity with 49 events, pooled analysis 4.5% (95% CI 1.1–7.8%, I^2^ = 97%) (Fig. 3d).

Pooled results of the two studies that reported cardiac data showed no difference in the odds of events between patients treated with RT and those who did not receive RT (24 events, pooled analysis 1.6% (95% CI 0.3–5%, I^2^ = 86%) (Fig. 3e).

Summary of Findings (SoF) tables for toxicity was reported in Table 2.

**Table 2:** Summary of findings (SoF) table for outcomes of harm.

Study design	Risk of bias	Inconsistency	Indirectness	Imprecision	N. of patients		Effect		Certainty
					PMRT	no PMRT	Relative (95% CI)	Absolute (95% CI)	
6 sudies; Adjuvant RT; acute grade 3–4 toxicity									
Observational	Not serious	Serious^a^	Not serious	Not serious	6 studies with 222 events; pooled analysis 11% (95% CI 6.1–17%). I^2:^97%				Very low
3 studies; Adjuvant RT; late grade 3–4 skin toxicity									
Observational	Not serious	Serious^b^	Not serious	Not serious	3 studies with 8 events; pooled analysis 0.5% (95% CI 0.1–1%). I^2^:61%				Very low
3 studies; Adjuvant RT; late grade 3–4 lung toxicity									
Observational	Not serious	Serious^a^	Not serious	Not serious	3 studies with 49 events; pooled analysis 4.5% (95% CI 1.1–7.8%). I^2^:97%				Very low
3 studies; Adjuvant RT; late grade 3 lymphedema									
Observational	Not serious	Serious^c^	Not serious	Not serious	3 studies with 24 events; pooled analysis 1.3% (95% CI 0–2.6%). I^2^:78%				Very low
2 studies; Adjuvant RT; cardiac events									
Observational	Not serious	Serious^d^	Not serious	Not serious	2 studies with 24 events; pooled analysis 1.6% (95% CI 0–3.5%). I^2^:86%				Very low

*Abbreviations:* RT: radiotherapy; HR: hazard ratio; CI: confidence interval; RR: risk ratio.

Explanations.

a I2:97%.

b I2:61%.

c I2:78%.

d I2:86%.

### 3.4 EtD (evidence to decision) framework

The proposed intervention (loco-regional radiation therapy) compared to the control (observation) increases, ‘per se’, the incidence of side effects, with a benefit that the poor reliability of the data makes not substantially evident. In the setting of patients with pN2 disease after breast surgery and axillary clearance, receiving postoperative chemotherapy, radiation therapy has proven effective in reducing local recurrences and possibly improving survival. It is also evident that the use of preoperative chemotherapy can select, based on the pathological response, the patients with the best prognosis, even if robust data on local control are missing.

These considerations make it difficult to suggest an postoperative strategy including radiation therapy for the patients included in the developed question, and suggests a case-by-case evaluation by the clinician based on the biological phenotype (i.e. luminal-like type vs triple negative, HER2-positive) and specific clinical characteristics (tumor extension at diagnosis and residual disease after preoperative treatment).

### 3.5 Beneﬁt/harm balance and final recommendation

The panel voted for the beneﬁt/harm beneﬁt as uncertain. The strength of the recommendation was voted as conditionally weak by 5 out of 5 panel members. Hence, the ﬁnal recommendation released by the panel was: in patients with cN2 at diagnosis turning ypN0 at surgery after preoperative chemotherapy, loco-regional RT may be at the discretion of the patient and clinician after extensive discussion preferably within a multidisciplinary team (Table 3).

**Table 3:** Final recommendation.

QUALITY OF EVIDENCE	RECOMMENDATION	STRENGTH OF RECOMMENDATION
VERY LOW	In patients with cN2 at diagnosis turning ypN0 at surgery after preoperative chemotherapy, post mastectomy radiation therapy (LCRT) may be at the discretion of the patient and clinician after extensive discussion preferably within a multidisciplinary team	Conditional both for intervention and comparator

## 4 Discussion

The AIRO Clinical Practice Guidelines on Breast Cancer panel cannot suggest the use of loco-regional RT in breast cancer patients with cN2 at diagnosis turning ypN0 after PST.

This is underlined by the lack of data on the role of postoperative radiation therapy after PST for breast cancer based on treatment response. While the prognostic impact of pCR after PST on DFS and OS has been shown in meta-analyses of randomized phase III trials [43], the association of treatment response with loco-regional recurrence has been studied only in retrospective reports [44].

Heterogeneity in treatment protocols, inclusion in the target volume of the IMN, and selection bias could affect the effective role of RNI in this setting of patients. The only powered analysis using sophisticated statistical methods to account for heterogeneities in patient characteristics [45] is compromised by missing details on the treatment fields and target volumes, as well as missing information on LRRFS and DFS. For all these reasons, the role of loco-regional RT in breast patients with cN2 at diagnosis and ypN0 after PST is controversial. Indeed, few studies showed as in patients with clinical stage III disease loco-regional RT seem to have a significant benefit even in the setting of a pCR [24] while others failed to show a benefit for RNI in this subgroup. However, some of these studies included a significant number of patients with clinically unsuspicious lymph nodes and were statistically underpowered. Due to the retrospective nature of the included studies, the quality of evidence is poor. Since several randomized controlled studies have shown a benefit of RNI in patients with limited or no nodal involvement and further risk factors treated with up-front surgery [46,47], RNI should be strongly considered in patients with clinically involved lymph nodes regardless of the response to PST, especially in the presence of further risk factors (e. g. young age, ER/PR-negative, lympho-vascular invasion, residual tumor in the breast).

In the present analysis loco-regional RT was found to be safe. Wang et al. [37] found grade 3 acute skin toxicity equal to 8% (32 patients) and symptomatic radiation pneumonitis occurring in less than 5% of patients. Late skin toxicity and symptomatic lung fibrosis were uncommon. Early grade 3 skin toxicity was observed in 4.5% (43 cases) and increased with concomitant chemotherapy (P < 0.001) and smoking (P = 0.06). Only three patients developed ischemic heart disease: all were treated with anthracycline-based CT with or without trastuzumab, and irradiated to the left chest wall and LN. They all presented numerous cardiovascular risk factors (2–4 factors). Another risk factor was the use of bolus in loco-regional RT which correlated with a higher rate of acute grade 2 skin toxicity [38,40]. Loco-regional treatment with IMRT technique can reduce the incidence rate of radiation toxicity by decreasing organs at risk (OARs) irradiation [37,39].

The current study has a number of limitations that deserve consideration. The number of included studies was small, especially for the secondary outcome measures, and all studies were non-randomized. This has implications on the results because non-randomized studies carry a number of inherent biases. Non-randomized trials do not use concealed randomization; hence, the groups may not be comparable, leading to selection bias. In addition, the presence of other confounding factors such as unadjusted background variables, detection, and recall bias due to selective reporting, or the presentation of incomplete outcome data, may affect the results of the analysis. These are all potential biases that may influence the validity of the study results. On the other hand, the quality assessment indicated that the studies were of moderate to high quality. No control for patient comorbidities was included in the analysis. Because of the small number of studies, important subgroup analyses could not be performed, e.g. clinic-pathologic factors, hormonal receptor status, and Her2/neu status, which are known to be associated with treatment efficacy, was missing in some patients. The toxicity data were extrapolated from retrospective studies with different outcomes and some with short follow-up. A median follow-up of 5 years is insufficient to allow for the assessment of all potential late toxicities. However, longer follow-up is unlikely to lead to the detection of further differences in late toxic effects between the two groups.

## 5 Conclusions

Because of the limitations due to the small number of studies and the heterogeneity in treated patients, the panel is not able to draw any definitive conclusions and highlights the need for well-controlled trials to determine the effect of RT in patients with cN2 and ypN0 after PST. The ﬁnal recommendation released by the panel was: in patients with cN2 at diagnosis and ypN0 at surgery after PST, loco-regional RT should be evaluated for each patient after extensive discussion in the multidisciplinary team. In discussion with patients, the following aspects should be highlighted:

• The prognosis with and without treatment;
• The limited evidence on the benefit derived from radiant treatment;
• The risks and benefits including late cardiac and pulmonary events and possible side effects on reconstruction, when offered.

## Funding

This research did not receive any specific grant from funding agencies in the public, commercial, or not-for-profit sectors.

## Declaration of competing interest

None.
